# Impacts of Hematopoietic Stem Cell Transplantation on Quality of Life and Behavioral Outcomes in Pediatric Patients with Leukemia and Thalassemia

**DOI:** 10.3390/curroncol32060311

**Published:** 2025-05-28

**Authors:** Aysun Kara Uzun, Sevilay Yıldız Akkuş, Ayça Koca Yozgat, Fadime Yüksel, Özlem Arman Bilir, Hüsniye Neşe Yaralı, Namık Yaşar Özbek

**Affiliations:** 1 Department of Pediatrics, University of Health Sciences, Ankara 06018, Turkey; ayca.kocayozgat@sbu.edu.tr (A.K.Y.); fadime.yuksel@sbu.edu.tr (F.Y.); ozlem.armanbilir@saglik.gov.tr (Ö.A.B.); namukyasar.ozbek@sbu.edu.tr (N.Y.Ö.); 2 Division of Social Pediatrics, Department of Pediatrics, Children’s Hospital, Ankara Bilkent City Hospital, Ankara 06800, Turkey; 3 Child Development Unit, Children’s Hospital, Ankara Bilkent City Hospital, Ankara 06800, Turkey; sevilay.yildiz4@saglik.gov.tr; 4 Department of Pediatric Hematology, Children’s Hospital, Ankara Bilkent City Hospital, Ankara 06800, Turkey; hnyarali@aybu.edu.tr; 5 Department of Pediatrics, Ankara Yıldırım Beyazıt University, Ankara 06010, Turkey

**Keywords:** behavior, hematopoietic stem cell transplantation, leukemia, quality of life, thalassemia

## Abstract

Background: This study aimed to investigate the effects of allogeneic hematopoietic stem cell transplantation (HSCT) on quality of life and behavioral problems in children diagnosed with leukemia and β-thalassemia major, with a focus on post-transplant diagnosis-specific differences in psychosocial adjustment. Method: This study included 112 children (63 children with acute leukemia, 49 children with β-thalassemia major) aged 6–18 years, along with a control group of 30 healthy children within the same age range. The Pediatric Quality of Life Inventory (PedsQL) and the Child Behavior Checklist for Ages 6–18 (CBCL) were administered. Participants were categorized into five groups, and the outcomes were compared accordingly. Results: The emotional functioning subscale scores of the PedsQL were significantly lower in children with leukemia and those who had undergone HSCT for leukemia, when compared to children with thalassemia (*p* < 0.05). The social functioning subscale scores were also significantly lower in children with leukemia who underwent HSCT compared to those with thalassemia (*p* < 0.05). The CBCL internalizing scores were higher in children with leukemia and post-HSCT leukemia patients than in their healthy peers (*p* < 0.05). Over time, both diagnostic groups showed improvements, with total PedsQL scores increasing and total CBCL scores decreasing after HSCT. Conclusions: This study demonstrates that quality of life improved and behavioral problems diminished over time following HSCT. However, the psychosocial impact of HSCT varied depending on the underlying disease, with children diagnosed with leukemia being slightly more adversely affected. These findings suggest that interventions aimed at improving quality of life and addressing behavioral issues should be tailored to the specific diagnosis.

## 1. Introduction

Chronic illnesses profoundly affect not only the lives of children, but also those of their families. For children diagnosed with hematological conditions such as leukemia and β-thalassemia major, their daily life undergoes a dramatic transformation. These diseases are characterized by prolonged and complex treatment regimens, which can substantially impact both the quality of life (QoL) of the child and family, as well as the child’s behavioral development [1,2].

At present, 60–70% of pediatric cancer patients achieve full remission. Given the high survival rates and extended life expectancy, aspects such as timely and effective treatment, access to optimal care, improved quality of life, and a holistic psychosocial approach have become increasingly significant. Advances in early diagnosis, novel chemotherapeutic protocols, and particularly hematopoietic stem cell transplantation (HSCT) have played pivotal roles in improving survival outcomes. HSCT remains a cornerstone in the treatment of pediatric leukemia and thalassemia [1,2].

β-thalassemia major, if left untreated or inadequately managed, leads to a markedly reduced life expectancy and severely diminished quality of life. It represents a significant public health concern in Mediterranean countries, including Turkey [3].

Behavioral problems in children are characterized by persistent patterns of maladaptive behaviors such as self- or other-directed aggression, excessive crying, oppositional conduct, dishonesty, destructiveness, and tantrums. Previous research has also identified emotional dysregulation, impulsivity, and poor conformity to social norms as key components of behavioral dysfunction [4,5]. In children with chronic illnesses, physical limitations and disease-related restrictions may foster the emergence of negative emotions and behavioral disturbances. Young children, particularly those of play age, may interpret physical discomfort and pain as punishment, contributing to maladaptive emotional responses [6]. The physical challenges during this developmental phase can impair motor control and hinder social competence, resulting in passivity, dependency, and social withdrawal [7]. Studies have shown that children with chronic illnesses are twice as likely to develop adjustment problems compared to their healthy peers, with the key contributing factors including perceived inadequacy and disease-related limitations [8].

The concept of quality of life is now regarded as a vital complement to traditional metrics of health and functionality. The World Health Organization defines health as a state of complete physical, psychological, and social well-being. In this context, comprehensive health assessments should encompass not only physical status but also psychological and social dimensions, including health-related quality of life (HRQoL) [9,10]. Recent advances in medical care have shifted attention toward QoL, particularly in scenarios where full recovery may not be achievable. Increasingly, the benefit of treatment is being evaluated not only in terms of survival, but also its capacity to enhance QoL [11].

In the existing literature, studies on the quality of life and behavioral problems of thalassemia or leukemia patients before and after HSCT have generally been conducted separately. Although both diseases are chronic, the time taken for HSCT is varied [12,13]. The rate of short- and long-term complications of thalassemia may be higher than in patients with acute leukemias. At the time of transplantation, in contrast to leukemia patients, the immune system of patients with thalassemia is not heavily impaired by previous cytotoxic agent therapy and, in this setting, the increased risk of complications is exacerbated by sensitization to RBC antigens and development of anti-HLA antibodies as a consequence of prolonged RBC transfusions. In particular, iron overload and the related hyper-production of reactive oxygen species, resulting from ineffective erythropoiesis and chronic transfusions, are associated with short- and long-term complications after HSCT [14].

Therefore, in this context, the necessity and content of the psychosocial approach may also vary. Consequently, these two diagnostic groups—as those most frequently treated with HSCT in our clinic—were selected for analysis synchronously. The present study aims to fill a gap in the existing literature through determining the differences in psychosocial needs between the considered groups, thereby supporting timely and targeted interventions.

## 2. Method

The study sample comprised children aged from 6 to 18 years diagnosed with leukemia or β-thalassemia major who were followed and treated at the Bone Marrow Transplantation and Hematology Services and Outpatient Clinics of Ankara Bilkent City Hospital, Children’s Hospital. Their families, who agreed to participate in the study were also included in the study. A total of 142 participants were enrolled between 1 December 2021, and 1 June 2022.

Participants were categorized into five groups: children diagnosed with leukemia more than six months previously, children with leukemia who had undergone HSCT more than six months previously, children diagnosed with β-thalassemia major more than six months previously, children with β-thalassemia major who had undergone HSCT more than six months previously, and a control group consisted of healthy children and adolescents in the same age range who had visited the pediatric outpatient clinics for routine check-ups, had no chronic illnesses, and reported no psychological, behavioral, or emotional complaints. It should be noted that the diagnostic groups with and without HSCT were not composed of the same children. Children that had been diagnosed with leukemia or thalassemia and/or undergone HSCT within six months previously were excluded, in order to minimize the confounding effects of acute treatment on behavior and quality of life.

Ethical approval was obtained from the Clinical Research Ethics Committee of Ankara Bilkent City Hospital prior to data collection (Decision No: E2-21-1033).

All participants completed an informed consent form, a general information form, the Pediatric Quality of Life Inventory (PedsQL), and the Child Behavior Checklist for Ages 6–18 (CBCL/6–18). These instruments were completed by the children’s mothers in a private room without external assistance, taking approximately 25 min.

The General Information Form, developed by the researchers, consisted of two sections: the first collected sociodemographic data (e.g., age, gender, number of siblings, parental age and education, school attendance, family structure); the second captured disease-specific data (e.g., diagnosis, age at diagnosis, length of hospitalization, age at HSCT, time elapsed since HSCT, frequency of follow-ups).

The Pediatric Quality of Life Inventory (PedsQL), developed by Varni et al. (1999), measures quality of life in children aged 2–18 and has been validated in Turkish by Üneri and Memik [15,16,17,18]. It includes parent-report forms and child self-report forms, with different versions for specific age groups. The parent-report form contains 23 items across four domains: physical, emotional, social, and school functioning. Items are rated on a 5-point Likert scale (3-point for ages 5–7), with the overall score ranging from 0 to 100, with higher scores indicating better quality of life. The Cronbach’s alpha for the parent version is 0.87.

The Child Behavior Checklist for Ages 6–18 (CBCL/6–18) is a widely used parent-report measure developed by Achenbach and Rescorla and adapted into Turkish by Erol et al. [19,20,21]. It assesses behavioral and emotional problems over the past six months using 113 items scored as 0 (not true), 1 (somewhat true), or 2 (very true). The scale yields internalizing and externalizing problem scores, as well as several subscales, including anxiety/depression, somatic complaints, social problems, thought problems, attention problems, rule-breaking, and aggression. The Turkish version demonstrates strong internal consistency (Cronbach’s alpha: 0.88) and test–retest reliability (r = 0.84).

### Data Analysis

All data were analyzed using the Statistical Package for Social Sciences (SPSS), version 25.0 (IBM Corp., New York, NY, USA, 2017). Categorical variables are summarized using frequencies and percentages, and continuous variables are presented as means and standard deviations. Normality of data distribution was assessed using skewness–kurtosis statistics and the Shapiro–Wilk test. Due to non-normal distributions and the presence of multiple groups, the Kruskal–Wallis H test was used to compare scores across groups. Statistical significance was defined as *p* < 0.05. When significant differences were found, pairwise comparisons were performed using the Bonferroni correction. In addition, this study aimed to examine the differences in scale scores according to disease groups (leukemia and β-thalassemia) and the time elapsed after HSCT. However, as a normal distribution was not validated, two-way ANOVA could not be performed. Instead, comparisons were made using the Kruskal–Wallis H test, both for the entire group (i.e., without dividing the disease groups) and for each disease group separately. In this case, in order to reduce the risk of Type I errors (false positives) that can occur when more than one statistical test is performed, the Bonferroni correction was implemented through dividing the statistical significance level by the number of comparisons. Statistical significance was accepted as 0.05/3 = 0.017 for these comparisons. In addition, effect sizes are reported for comparisons where statistically significant differences were detected, in order to demonstrate the effectiveness of the detected difference. As the Kruskal–Wallis H test—a non-parametric test—was used, the rank eta squared value is reported as the measure of effect size. Effect size interpretation values commonly cited in the published literature are as follows: 0.01 to less than 0.06 indicates a small effect; 0.06 to less than 0.14 indicates a moderate effect; and values equal to or greater than 0.14 indicate a large effect.

## 3. Results

All 142 participants completed the study forms in full, and there were no missing data. The study group included 142 children: 32 (22.5%) children with leukemia who underwent HSCT, 20 (14.1%) children with thalassemia who underwent HSCT, 31 (21.8%) children with leukemia, 29 (20.4%) children with thalassemia, and 30 (21.1%) healthy controls. Among the children with acute leukemia, 10 were diagnosed with acute myeloid leukemia and 53 with acute lymphoblastic leukemia. All children with thalassemia were diagnosed with β-thalassemia major, and all transplanted children had received a single allogeneic HSCT.

Of the children participating in this study, 49.3% (70) were girls and 50.7% (72) were boys. The mean age of the children was 12.14 years.

Statistically significant differences were observed in the PedsQL scores for emotional and social functioning and total scores across disease groups (*p* < 0.05; Table 1). The significant difference obtained for emotional functionality according to disease groups had a moderate effect size ($\eta^{2}$ = 0.066), while the significant differences obtained for social functioning and total scores according to disease groups had a small effect size ($\eta^{2}$ = 0.058 and 0.059, respectively). Specifically, emotional functioning scores were significantly higher in children with thalassemia, compared to those with leukemia (*p* = 0.019) and those with leukemia who underwent HSCT (*p* = 0.031). Similarly, children with thalassemia demonstrated significantly higher social functioning scores than those with leukemia who had undergone HSCT (*p* = 0.020).

Regarding CBCL scores, significant differences were observed in internalizing, externalizing, and total scores across disease groups (*p* < 0.05; Table 2). The significant difference obtained for internalizing and externalizing according to disease groups had a moderate effect size ($\mathrm{respectively},\;\eta^{2}$ = 0.074; 0.092), while the significant differences obtained for total scores according to disease groups had a small effect size ($\eta^{2}$ = 0.058). Pairwise comparisons showed that internalizing scores were significantly higher in children with leukemia (*p* = 0.015) and those with leukemia post-HSCT (*p* = 0.009), compared to the control group. For externalizing behaviors, children with leukemia scored significantly higher than controls (*p* = 0.031). Similarly, total CBCL scores were significantly higher in children with leukemia (*p* = 0.002) and leukemia post-HSCT (*p* = 0.033), compared to controls.

A statistically significant difference was found in PedsQL physical functioning scores based on the time elapsed since HSCT (*p* < 0.017), which was characterized by a large effect size ($\eta^{2}$ = 0.600; Table 3). Post hoc analysis revealed significant differences between the 6–12-month group and both the 19–24-month (*p* = 0.001) and >2-year groups (*p* = 0.000), as well as between the 13–18-month and >2-year groups (*p* = 0.006). When stratified by disease, both diagnostic groups showed significant changes in physical functioning over time post-HSCT (*p* < 0.017), both with a large effect size ($\eta^{2}$ = 0.593 for the leukemia group; $\eta^{2}$ = 0.682 for the thalassemia group). Post hoc analysis for children with leukemia revealed significant differences between the 6–12-month group and both the 19–24-month (*p* = 0.007) and >2-year groups (*p* = 0.000). Post hoc analysis for children with thalassemia revealed significant differences between the >2-year groups and both the 6–12-month (*p* = 0.019) and 13–18-month groups (*p* = 0.019). When the median values were examined in general, it was seen that there was an increase in PedsQL physical functioning scores in both groups according to the time elapsed after HSCT. The PedsQL physical functioning scores for children with leukemia and thalassemia were closer at 6–18 months after HSCT. On the other hand, at 19–24 months and >2 years after HSCT, the increases in PedsQL physical functioning scores for children with leukemia were greater than those in children with thalassemia.

For the PedsQL emotional subscale, significant time-dependent differences were observed (*p* < 0.017), with a large effect size ($\eta^{2}$ = 0.533); particularly between the 6–12-month group and both the 19–24-month (*p* = 0.000) and >2-year groups (*p* = 0.000). Within diagnostic subgroups, children with leukemia who underwent HSCT showed significant differences (*p* < 0.017), with a large effect size ($\eta^{2}$ = 0.707), whereas those with thalassemia did not (*p* > 0.017). Post hoc analysis for children with leukemia revealed significant differences between the 6–12-month group and both the 19–24-month (*p* = 0.000) and >2-year (*p* = 0.001) groups. When the median values were examined in general, the PedsQL emotional subscale scores were higher for children with thalassemia than those with leukemia at 6–18 months after HSCT, while the scores were higher for children with leukemia than those with thalassemia at 19–24 months and >2 years.

The social functioning scores in the PedsQL also varied significantly with the time since HSCT (*p* < 0.017), presenting a large effect size ($\eta^{2}$ = 0.524). Significant differences were found between the 6–12-month and 19–24-month (*p* = 0.013) and >2-year (*p* = 0.000) groups, as well as between the 13–18-month and >2-year groups (*p* = 0.025). Both disease groups exhibited significant differences in social functioning over time (*p* < 0.017), with a large effect size ($\eta^{2}$ = 0.472 for the leukemia group; $\eta^{2}$ = 0.599 for the thalassemia group). Post hoc analysis for children with leukemia revealed significant differences between the 6–12-month group and both the 19–24-month (*p* = 0.037) and >2-year (*p* = 0.000) groups. Post hoc analysis for children with thalassemia revealed significant differences between the 6–12-month group and >2-year groups (*p* = 0.006). When the median values were examined in general, the PedsQL social functioning scores were higher for children with thalassemia than those with leukemia at 6–18 months after HSCT, and higher for children with leukemia than those with thalassemia at 19–24 months and >2 years.

In the PedsQL school functioning subscale, time since HSCT was also a significant factor (*p* < 0.017), presenting a large effect size ($\eta^{2}$ = 0.231); in particular, there was a notable difference between the 6–12-month and >2-year groups (*p* = 0.001). However, within the disease groups, no significant differences were found over time (*p* > 0.017). When the median values were examined in general, the PedsQL school functioning subscale was found to be higher for children with thalassemia than those with leukemia at 6–24 months after HSCT, and higher for children with leukemia than those with thalassemia at >2 years after HSCT.

**Figure 1:**
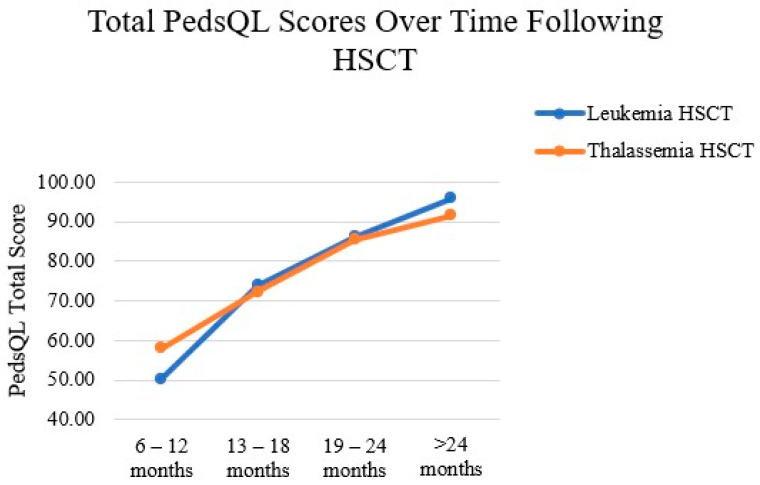
Means of total PedsQL scores by time since HSCT in children with leukemia and thalassemia.

Total PedsQL scores increased significantly with the time elapsed since HSCT (*p* < 0.017), presenting a large effect size ($\eta^{2}$ = 0.790). Differences were evident between the 6–12-month and both the 19–24-month (*p* = 0.000) and >2-year (*p* = 0.000) groups, as well as between the 13–18-month and >2-year groups (*p* = 0.001). Both diagnostic groups demonstrated significant improvements in total PedsQL scores over time (*p* < 0.017), both with a large effect size ($\eta^{2}$ = 0.792 for the leukemia group; $\eta^{2}$ = 0.842 for the thalassemia group). Post hoc analysis for children with leukemia revealed significant differences between the 6–12-month group and both the 19–24-month (*p* = 0.001) and >2-year (*p* = 0.000) groups. Post hoc analysis for children with thalassemia revealed significant differences between the 2-year group and both the 6–12-month (*p* = 0.003) and 13–18-month (*p* = 0.025) groups. In addition, as shown in Figure 1, the mean total PedsQL inventory scores increased over time in both diagnostic groups following HSCT. PedsQL total scores were higher for children with thalassemia than those with leukemia at 6–12 months after HSCT; the scores in the two groups were almost the same at 13–24 months after HSCT; and higher scores were observed for children with leukemia than those with thalassemia at >2 years after HSCT.

CBCL internalizing scores also showed a significant decline over time (*p* < 0.017), with a large effect size ($\eta^{2}$ = 0.428; Table 4); this was particularly evident between the >2-year group and both the 13–18-month (*p* = 0.037) and 6–12-month (*p* = 0.000) groups. When analyzed by diagnosis, children with leukemia post-HSCT exhibited a significant improvement (*p* < 0.017), with a large effect size ($\eta^{2}$ = 0.417); meanwhile, no such difference was observed in children with thalassemia (*p* > 0.017). Post hoc analysis for children with leukemia revealed significant differences between the 6–12-month and >2-year groups (*p* = 0.000).

No significant differences were detected in externalizing behavior scores with the time since HSCT (*p* > 0.05), either overall or within individual diagnostic groups.

**Figure 2:**
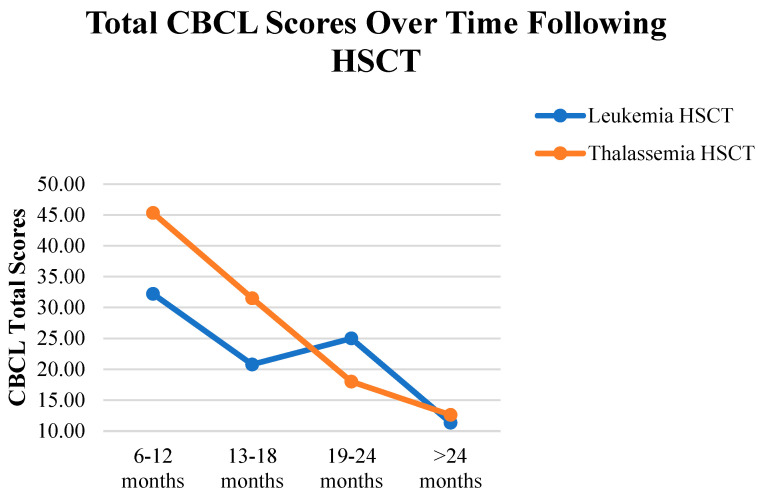
Means of total CBCL scores by time since HSCT in children with leukemia and thalassemia.

Finally, the CBCL total scores varied significantly based on the time since HSCT (*p* < 0.017), with a large effect size ($\eta^{2}$ = 0.248); in particular, a notable reduction in scores was observed among children who had undergone HSCT > 2 years earlier, compared to those at 6–12 months (*p* = 0.001). Median scores indicated lower total CBCL scores in the >2-year group. Meanwhile, no significant changes in total CBCL scores were found over time in the leukemia (*p* > 0.017) and thalassemia (*p* > 0.017) groups. In addition, as shown in Figure 2, the mean total CBCL scores decreased over time in both diagnostic groups following HSCT.

## 4. Discussion

Hematopoietic stem cell transplantation is a life-saving intervention. In the management of chronic illnesses, healthcare professionals and families often prioritize the underlying disease, while the child’s QoL and psychosocial health may be overlooked. HSCT can significantly impact these domains, making it essential to raise awareness and implement early interventions to support affected children and families.

This study investigated the impacts of HSCT on QoL and behavioral problems in children diagnosed with leukemia and β-thalassemia major, with a focus on diagnosis-specific differences in post-transplant psychosocial adjustment, as well as how these effects evolve over time post-transplantation.

The findings presented in this study indicate that both diagnostic groups—with or without HSCT—exhibited generally lower PedsQL scores and greater behavioral problems when compared to healthy peers. However, over time, improvements were observed in QoL and reductions in behavioral symptoms following HSCT. The work of van Litsenburg et al. reported that QoL scores were affected negatively in children with leukemia [22]. Liang et al. reported that children with thalassemia between the ages of 5 and 18 had lower scores than their healthy peers in the PedsQL physical functioning score, school functioning subscale, and total scores [23]. Leukemia treatment affects daily activities, family life, peer relationships, and school success, leading to a low QoL. Physical sequelae and late side effects after transplantation have also been shown to reduce QoL [24,25].

In this study, emotional functioning, social functioning, and total PedsQL scores varied significantly by disease type, whereas physical and school functioning did not. The absence of differences in physical and school functioning by disease type may be attributed to the inclusion criteria of participants, in that the period within the first 6 months after diagnosis and transplantation was not evaluated.

Emotional functioning scores were lower in children with leukemia and those who had undergone HSCT for leukemia compared to those with thalassemia. Similarly, children with leukemia post-HSCT presented lower social functioning scores than those with thalassemia. Chronic diseases and their intensive treatment regimens, including HSCT, can negatively affect both QoL and psychosocial development. Post-HSCT complications—such as immune suppression, graft-versus-host disease, secondary malignancies, growth and developmental issues, and infertility—can contribute to diminished QoL. During the early post-transplant period, children are often isolated and removed from normal activities, which can exacerbate the physical, psychological, and social challenges that they face [22,24,25,26].

The relatively better emotional and social functioning scores observed in children with thalassemia may reflect their earlier age at diagnosis and greater adaptability to long-term care processes. Moreover, differences in treatment intensity and follow-up frequency between leukemia and thalassemia patients may account for these outcomes. Children with leukemia may experience heightened vulnerability due to intensive treatments and frequent monitoring for late-onset complications. Clarke et al. similarly reported significantly lower HRQoL scores in children with acute leukemia post-HSCT, when compared to non-transplanted patients and healthy controls [27].

This study also found significant differences in CBCL internalizing, externalizing, and total scores across disease groups. Children with leukemia and those who underwent HSCT had higher internalizing scores than their healthy peers, while externalizing scores were higher in the leukemia group. Total CBCL scores were elevated in both the leukemia and leukemia post-HSCT groups. A majority of leukemia patients post-HSCT (81.2%) were within 6–24 months post-transplant—a period during which psychosocial recovery may still be ongoing. At the same time, it was determined that children with leukemia may display externalizing behaviors (e.g., aggressive behaviors and rule-breaking) in the early stages of diagnosis. These findings may be explained by the older age at diagnosis and shorter duration since diagnosis among the leukemia patients, as well as the perceptions of the illness among the children and their families. The results underscore the substantial psychosocial burden of leukemia and the need for tailored support post-transplantation. There was no difference in CBCL scores between children with thalassemia/children with thalassemia who underwent HSCT and their healthy peers, which may be due to the earlier age of diagnosis in children with thalassemia and their better adaptation to long-term care processes. Prior research has indicated that problem behavior scores tend to decline as time passes after HSCT [24]. Similarly, Clarke et al. found that children with leukemia exhibited more behavioral problems, as measured using the Strengths and Difficulties Questionnaire, at one year or more post-HSCT [27].

In the two disease groups, our findings revealed that QoL (according to the PedsQL) was lowest at 6–12 months post-HSCT, followed by steady improvement over the next two years across all subdomains. This is consistent with the findings of Caocci et al., who reported progressive improvements in QoL from 3 to 6 months to 18 months post-HSCT in children with thalassemia major [28]. In another study, children who had undergone HSCT four or more years earlier reported QoL levels similar to healthy controls and better than those of non-transplanted patients in all subdomains [23]. Although the PedsQL emotional subscale scores were better in children with thalassemia in the early stages of HSCT, they did not differ much over time following HSCT when compared to children with leukemia. Compared with children with thalassemia, the improvement in the emotional status of children with leukemia became evident at 19 months after transplantation; furthermore, it was determined that they were in a better condition than children with thalassemia at two years after transplantation. This result reveals that psychosocial support approaches should be planned according to the type of disease, and should be continued for a long time in both disease groups. Considering the results of the PedsQL school functioning subscale in this study, educational planning for children in the early period of HSCT and in the long-term should be specifically designed according to the disease group.

It is known that children who undergo HSCT during childhood and adolescence are at risk of developing social and behavioral problems in the long-term [29]. Children and adolescents are more vulnerable during illness and treatment in terms of cognitive, perceptual, educational, motor skills, and psychosocial aspects [30]. Children are kept away from their schools and friends in the first few months after HSCT. During this period, there is also a decrease in their physical function due to the effects of the treatment. It is known that physical isolation increases depressive symptoms. Thus, children may experience emotional and behavioral problems when they return to their social activities and school after hospital discharge [31]. In this study, the CBCL total scores improved within the first two years post-transplantation. However, the greatest improvement was achieved at >2 years after HSCT. Internalizing scores in children with thalassemia did not differ much over time since HSCT, when compared to those with leukemia. It can be understood, from the study results, that externalizing behavioral changes are not seen very often in either disease group after HSCT. This result reveals that psychosocial support approaches should be planned according to the type of disease, and should be continued for a long time in both disease groups. Barrera et al. observed that problem behaviors, as measured according to the CBCL, decreased significantly at six months after HSCT in a cohort where over half of the participants were children with leukemia [24].

This study has several limitations. The diagnostic groups with and without HSCT were not composed of the same children, and the time elapsed since transplantation varied across participants. Although leukemia and thalassemia are both chronic diseases, the time taken for HSCT varies [12,13]. It would have been better if the study controlled for confounding variables such as age, socioeconomic status, and parental education, but because of the low number of cases, the groups would be smaller when confounding factors were evaluated, and the results would become undefined. Prospective studies with larger samples and controlled confounding factors are needed. Furthermore, only parent-report forms of the PedsQL and CBCL were used; child self-reports were not included, which may limit insight into the children’s perceptions. Bias may be introduced when depending solely on maternal reports. Nonetheless, this study benefits from its focus on a single treatment center and inclusion of a well-matched healthy control group. Importantly, it is among the few studies to assess the impacts of HSCT on QoL and behavioral problems separately for children with leukemia or thalassemia.

## 5. Conclusions

Improvements were observed in QoL and behavioral outcomes across both disease groups over time. Children with leukemia had lower PedsQL emotional and social functioning subscale scores, and internalizing and externalizing behavioral scores were higher in children with leukemia when compared to those with thalassemia. There is a need to plan psychosocial support approaches according to the type of disease, and psychosocial support should be promptly provided to children with leukemia after diagnosis and in the early period of HSCT. While the QoL and CBCL results of children with thalassemia were better in the early period of HSCT, the results for children with leukemia improved with increasing time after transplantation. Long-term monitoring and psychosocial support are needed to ensure and improve the well-being of children with thalassemia and leukemia. Early identification of behavioral problems and targeted interventions may enhance the children’s recovery, prevent the worsening of behavioral issues, and alleviate parental concerns.

## Figures and Tables

**Table 1:** The results of the PedsQL inventory scores of participants.

PedsQL Inventory	Disease Groups	n	Median	IQR	H (df = 4)	*p*	$\eta^{2}$
Physical Functionality	Leukemia HSCT	32	78.13	42.19	5.076	0.280	-
	Thalassemia HSCT	20	85.94	32.81			
	Leukemia	31	71.88	25.00			
	Thalassemia	29	84.38	37.50			
	Control	30	93.75	26.56			
Emotional Functionality	Leukemia HSCT	32	77.50	37.50	13.084 *	0.011	0.066
	Thalassemia HSCT	20	90.00	21.25			
	Leukemia	31	75.00	25.00			
	Thalassemia	29	100.00	25.00			
	Control	30	90.00	18.75			
Social Functionality	Leukemia HSCT	32	80.00	27.50	11.894 *	0.018	0.058
	Thalassemia HSCT	20	90.00	15.00			
	Leukemia	31	80.00	30.00			
	Thalassemia	29	100.00	15.00			
	Control	30	95.00	10.00			
School Functionality	Leukemia HSCT	32	77.50	32.50	7.173	0.127	-
	Thalassemia HSCT	20	85.00	15.00			
	Leukemia	31	75.00	47.50			
	Thalassemia	29	90.00	35.00			
	Control	30	95.00	15.00			
Total	Leukemia HSCT	32	75.00	33.42	12.044 *	0.017	0.059
	Thalassemia HSCT	20	86.96	15.49			
	Leukemia	31	71.74	19.57			
	Thalassemia	29	89.13	28.26			
	Control	30	92.94	19.29			

* *p* < 0.05; n: number of participants; IQR: interquartile range; H: statistic of Kruskal–Wallis H test; $\eta^{2}$: rank eta-kare.

**Table 2:** The results of the CBCL scores of participants.

PedsQL Inventory	Disease Groups	n	Median	IQR	H (df = 4)	*p*	$\eta^{2}$
Internalizing	Leukemia HSCT	32	7.00	6.00	14.098 *	0.007	0.074
	Thalassemia HSCT	20	5.00	3.75			
	Leukemia	31	8.00	5.50			
	Thalassemia	29	6.00	4.00			
	Control	30	3.00	4.00			
Externalizing	Leukemia HSCT	32	4.50	9.50	11.921 *	0.018	0.058
	Thalassemia HSCT	20	4.50	5.00			
	Leukemia	31	5.00	6.50			
	Thalassemia	29	3.00	4.00			
	Control	30	2.00	4.00			
Total	Leukemia HSCT	32	19.50	24.00	16.550 *	0.002	0.092
	Thalassemia HSCT	20	17.50	11.50			
	Leukemia	31	23.00	20.00			
	Thalassemia	29	13.00	13.00			
	Control	30	9.50	14.00			

* *p* < 0.05; n: number of participants; IQR: interquartile range; H: statistic of Kruskal–Wallis H test; $\eta^{2}$: rank eta-kare.

**Table 3:** The results of the PedsQL inventory scores by the time elapsed after the HSCT procedure.

PedsQL Inventory	Time Elapsed After the HSCT	Leukemia			Thalassemia			Comparison for		
		n	Median	IQR	n	Median	IQR	Total Group	Children with Leukemia	Children with Thalassemia
Physical Functionality	6–12 months	9	53.13	21.88	3	43.75	18.75	H = 31.790 * p = 0.000 $\eta^{2}$ = 0.600	H = 19.618 * p = 0.000 $\eta^{2}$ = 0.593	H = 13.910 * p = 0.003 $\eta^{2}$ = 0.682
	13–18 months	9	68.75	18.75	4	54.69	10.16			
	19–24 months	8	92.19	14.06	3	87.50	3.13			
	Over 2 years	6	100.00	2.34	10	93.75	11.72			
Emotional Functionality	6–12 months	9	55.00	10.00	3	70.00	10.00	H = 28.594 * p = 0.000 $\eta^{2}$ = 0.533	H = 22.792 * p = 0.000 $\eta^{2}$ = 0.707	H = 4.983 *p* = 0.173
	13–18 months	9	70.00	15.00	4	87.50	26.25			
	19–24 months	8	97.50	6.25	3	90.00	5.00			
	Over 2 years	6	97.50	12.50	10	92.50	16.25			
Social Functionality	6–12 months	9	55.00	25.00	3	60.00	7.50	H = 28.133 * p = 0.000 $\eta^{2}$ = 0.524	H = 16.220 * p = 0.001 $\eta^{2}$ = 0.472	H = 12.587 * p = 0.006 $\eta^{2}$ = 0.599
	13–18 months	9	80.00	5.00	4	82.50	8.75			
	19–24 months	8	92.50	21.25	3	80.00	5.00			
	Over 2 years	6	100.00	3.75	10	95.00	5.00			
School Functionality	6–12 months	9	50.00	45.00	3	75.00	20.00	H = 14.111 * p = 0.000 $\eta^{2}$ = 0.231	H = 7.792 *p =* 0.051	H = 7.402 *p =* 0.060
	13–18 months	9	75.00	20.00	4	90.00	31.25			
	19–24 months	8	65.00	33.75	3	80.00	5.00			
	over 2 years	6	92.50	12.50	10	90.00	3.75			
Total	6–12 months	9	51.09	4.35	3	56.52	3.26	H = 40.924 * p = 0.000 $\eta^{2}$ = 0.790	H = 25.175 * p = 0.000 $\eta^{2}$ = 0.792	H = 16.472 * p = 0.001 $\eta^{2}$ = 0.842
	13–18 months	9	72.83	8.70	4	72.28	14.67			
	19–24 months	8	87.50	7.07	3	85.87	0.54			
	Over 2 years	6	97.28	6.79	10	91.30	4.35			

* *p* < 0.017; n: number of participants; IQR: interquartile range; H: statistic of Kruskal–Wallis H test; $\eta^{2}$: rank eta-kare.

**Table 4:** The results of the CBCL scores by the time elapsed after the HSCT procedure.

PedsQL Inventory	Time Elapsed After the HSCT	Leukemia			Thalassemia			Comparison for		
		n	Median	IQR	n	Median	IQR	Total Group	Children with Leukemia	Children with Thalassemia
Internalizing	6–12 months	9	14.00	10.00	3	21.00	11.00	H = 23.522 * p = 0.000 $\eta^{2}$ = 0.428	H = 14.683 * p = 0.002 $\eta^{2}$ = 0.417	H = 7.116 *p =* 0.068
	13–18 months	9	9.00	4.00	4	6.00	6.25			
	19–24 months	8	6.50	5.50	3	9.00	2.50			
	Over 2 years	6	4.00	0.75	10	3.50	3.75			
Externalizing	6–12 months	9	4.00	10.00	3	7.00	7.50	H = 2.020 *p =* 0.568	H = 1.213 *p =* 0.750	H = 2.502 *p =* 0.475
	13–18 months	9	5.00	8.00	4	5.50	5.25			
	19–24 months	8	6.00	10.25	3	2.00	3.00			
	Over 2 years	6	3.50	7.50	10	3.00	4.75			
Total	6–12 months	9	35.00	24.00	3	44.00	23.00	H = 14.909 * p = 0.002 $\eta^{2}$ = 0.248	H = 7.508 *p =* 0.057	H = 8.690 * p = 0.034 $\eta^{2}$ = 0.356
	13–18 months	9	16.00	15.00	4	20.50	17.00			
	19–24 months	8	23.00	19.25	3	21.00	6.50			
	Over 2 years	6	9.00	13.50	10	13.00	9.50			

* *p* < 0.017; n: number of participants; IQR: interquartile range; H: statistic of Kruskal–Wallis H test; $\eta^{2}$: rank eta-kare.

## Data Availability

The authors confirm that the data of the study is available in an electronic repository and will be shared upon request.
